# Incidence and risk factors for suicidal ideation in a sample of Chinese patients with mixed cancer types

**DOI:** 10.1007/s00520-022-07386-8

**Published:** 2022-10-21

**Authors:** Qianlin Lai, Hong Huang, Yinting Zhu, Siwei Shu, Yaner Chen, Yuanyuan Luo, Lili Zhang, Zhihui Yang

**Affiliations:** School of Nursing, Southern Medical University, No. 1023, South Shatai Road, Baiyun District, Guangzhou, 510515 Guangdong China

**Keywords:** Suicidal ideation, Cancer, Chinese, Demoralization

## Abstract

**Purpose:**

Suicidal ideation (SI) is often overlooked as a risk factor for people with cancer. Because it is often a precursor for suicidal behavior, it is critical to identify and address SI in a timely manner. This study investigated SI incidence and risk factors in a cohort of Chinese patients with mixed cancer types.

**Methods:**

Data from this cross-sectional study were collected from 588 patients receiving medical therapy for tumors at Nanfang Hospital and the Integrated Hospital of Traditional Chinese Medicine at Southern Medical University. SI was measured using the Self-rating Idea of Suicide Scale (SIOSS). Anxiety and depression were assessed using the Hospital Anxiety and Depression Scale (HADS). The Chinese version of the Demoralization Scale II (DS-II-C) was used to assess demoralization. Univariate and correlation analyses were used to identify correlative factors of SI and multiple stepwise linear regression analysis was used to characterize potential risk factors.

**Results:**

SI was reported in 24.7% of participants and the SIOSS score was 14.00 (13.00, 15.00) in the SI group. Multiple linear regression results showed that demoralization, medical financial burden, cancer type, living condition, caretaker, working state, residence, gender, and marital status explained 32.1% of the SI in this cohort (*F* = 28.705, *P* < 0.001).

**Conclusion:**

Approximately one-quarter of cancer patients in this study reported SI influenced by both external and internal factors. Characterizing these factors can be informative for prevention and treatment efforts.

## Introduction

China is a populous country with a high burden of cancer. In 2020, China registered 4.57 million new cases and 3 million deaths, ranking first worldwide [1]. Cancer is currently the second leading cause of death and is predicted to be the first cause in 2026 [2]. However, there are multiple causes of death for cancer patients. For example, several studies have reported that cancer patients are at particularly high risk of suicide. A retrospective, population-based study indicated that the rate of suicide per 100,000 person-years was 28.58, and the standard mortality ratio (SMR) for suicide was 4.44 [3]. A study conducted in the United States (U.S.) found that suicide was 27.5 per 100,000 person-years among cancer patients [4]. Indeed, patients with cancer are at a substantially higher risk of suicide than healthy people [5]. Cancer is a stressful event associated with physical and psychological pain and discomfort.

Suicidal behavior includes suicidal ideation (SI) and suicidal planning followed by suicide [6, 7]. SI involves thinking about suicidal ideas or plans and is an important predictor of suicide [8]. Timely detection and appropriate intervention can reduce the occurrence of suicide deaths, suggesting that SI is an important research topic. Factors associated with the risk of SI in cancer patients have been defined and include being unmarried or single [9]. Those with cancers of the lung, head and neck, gastrointestinal, and pancreas experience a higher risk of SI [10]. Depression, anxiety, and demoralization are also important psychological risk factors linked to SI among cancer patients [11–14].

Research on suicide in China has developed gradually since the 1880s [15]. To date, few studies have measured the incidence of SI and its risk factors among cancer patients in China. The present study investigated the incidence of SI among Chinese cancer patients and focused primarily on identifying both internal and external risk factors and assessing how each factor independently influences SI.

## Methods

### Study setting and participants

This cross-sectional study was conducted in the oncology departments in Nanfang Hospital and the Integrated Hospital of Traditional Chinese Medicine at Southern Medical University from 2020 to 2021. Both hospitals are tertiary A general hospitals affiliated with Southern Medical University.

All included participants met the following criteria: (1) a clinical diagnosis of cancer, (2) a minimum age of ≥ 18 years, (3) absence of cognitive impairment, and (4) provided signed informed consent. Exclusion criteria included those with (1) a medical history of mental illness or taking anti-psychotic drugs the past two weeks, (2) an inability to understand written or oral communication, and (3) a pre-existing psychological illness diagnosis in their medical record.

### Data collection and instruments

The research questionnaire consisted of a sociodemographic survey, the Self-rating Idea of Suicide Scale (SIOSS), the Hospital Anxiety and Depression Scale (HADS), and the Chinese version of the Demoralization Scale (DS-II-C). The sociodemographic questions included age (year), gender (male, female), level of education (primary and below, junior high school, senior high school, junior college, bachelor’s degree, or above), marital status (married, single, divorced, or widowed), caretaker (family member, nursing workers, friends, oneself), working state (still working, sick rest), income (< 3000, 3000–5000, > 5000 yuan), medical financial burden (not at all, a little, some, extreme), residence (rural, urban), living condition (not live alone, solitary), and cancer type (nasopharyngeal cancer, cholangiocarcinoma, lung cancer, liver cancer, colorectal cancer, lymphoma, ovarian cancer, breast cancer, esophageal cancer, stomach cancer, thymus cancer, pancreatic cancer, cervical cancer, other cancer).

#### SIOSS

The SIOSS was used to determine SI. The scale was created by the Chinese scholar Xia in 2001 and is based on the Beck Depression Inventory (BDI), the Symptom Checklist 90 (SCL-90), and the Minnesota Multiphasic Personality Inventory (MMPI) for screening SI. The scale consists of 26 items including desperation, optimism, sleep, and dissimulation factors that are scored with “yes” or “no” answers. An example item from the SIOSS reads as follows: “Do you want to end your life?” If the dissimulation factors totaled ≥ 4, the evaluation was considered invalid. A summed score ≥ 12 was the cut-off value for identifying patients with SI according to the evaluation method of SIOSS scale [16]. The higher the score, the stronger the SI. The Chinese version of the SIOSS has been used in multiple studies with different study populations and has strong reliability (*α* = 0.79) and validity [16–18]. The Cronbach’s *α* coefficient of the SIOSS for this study was 0.67.

#### HADS

The HADS is an assessment scale created by Zigmond and Snaith in 1983 and is primarily used to assess non-psychotic anxiety and depression symptoms among hospitalized patients [19]. The scale consists of two 7-item subscales used to evaluate anxiety and depression. Each item is rated on a 4-point scale of frequency ranging from 0 to 3 giving a maximum score of 21 for each subscale. An example of one of the items is: “Did you feel nervous or miserable during the past week?” Scores of 0–7 represent ‘asymptomatic’, 8–10 represent ‘borderline’, 11–14 represent ‘moderate’, and 15–21 represent ‘definitively positive.’ The reliability (*α* = 0.85) and validity of the scale have been previously verified [20]. The Cronbach’s *α* coefficient of the HADS for this study was 0.85.

#### DS-II-C

Demoralization is a maladaptive coping state characterized by hopelessness and helplessness and associated with a loss of purpose and meaning in life [21]. A simplified evaluative scale was adapted from the original demoralization scale created by Kissane et al. in 2016 [22]. DS-II-C is the Chinese adaptation of the simplified version and can be used to measure demoralization in cancer patients [23]. The scale includes 16 items divided into two subscales: (1) meaning and goals and (2) stress and coping subscales. Items on the Chinese original English versions are identical. Each item is based on a 3-point Likert scale (0 = never; 1 = sometimes; 2 = often) and higher scores are associated with higher levels of demoralization. One example item of the DS-II-C is that “Did you feel your life seems meaningless over the past 2 weeks?” Robinson divided the scale into three levels: low demoralization (< 25% of the full score), moderate demoralization (25–75% of the full score), and high demoralization (> 75% of the full score). The original DS is shown to have strong reliability (*α* = 0.94) and validity and the Chinese version also obtains wide application [23, 24]. The Cronbach’s *α* coefficient of the DS-II-C in this study was 0.92.

### Statistical analysis

Two researchers were responsible for creating a Microsoft Office Excel database and entering and maintaining the data. All analyses were performed using IBM SPSS Statistics for Windows, version 25.0 (IBM Corp., Armonk, NY, USA). Categorical data were expressed as frequency (*N*) and percentage (%) and continuous variables that conformed to the normal distribution were presented as the mean and standard deviation, otherwise median and interquartile range [*M* (Q25, Q75)]. The Mann–Whitney test was used for comparisons between two groups when the samples were non-normally distributed and the Kruskal–Wallis test was used for comparisons between multiple groups. Spearman’s correlation analysis was used to determine correlations associated with age, DS-II-C score, HADS score, and SIOSS score. Collinearity diagnosis was determined prior to multiple linear regression analysis. If the variance inflation factor (VIF) was > 5–10, multiple linear regression analysis was not performed. Variables with statistical differences in univariate and correlation analysis were included in the multiple stepwise linear regression equation for further statistical analysis. Continuous variables were input as raw data. Ordinal categorical variables were assigned, and nominal variables were set as dummy variables. A *P* value < 0.05 was considered statistically significant.

## Results

### Prevalence of SI

Of the 600 participants who received questionnaires, 588 were enrolled in the study. Six were excluded because their survey was incomplete or never returned and another six were excluded due to a high level of missing data. Thus, the questionnaire recovery and completeness rates were both 99.0%. A total score of 12 was set as the cut-off value for identifying patients with SI. Thus, the results of the SIOSS indicated that 145 survey participants had self-reported SI, while 443 did not. The SI prevalence in this sample of Chinese cancer patients was 24.7%.

### Descriptive analysis

Results of the descriptive analysis are shown in Table 1. The participants were 57.00 (50.00, 65.00) years of age on average and 59.5% were men. One-third (34.0%) of the participants had received a primary education or below, 93.0% were married, and only 10.7% held a religious belief. Most patients did not live alone (93.0%) and 55.3% lived in an urban area. Of the respondents, 50.5% had a monthly income of 3000–5000 yuan. Only 6.3% of participants experienced no medical financial burden. The primary types of cancer diagnosed were lung cancer (32.0%) and colorectal cancer (23.8%). The average SIOSS score in the SI group was 14.00 (13.00, 15.00), which was nearly twice as high as the average score in the NSI group. The average demoralization score in the SI group was 18.00 (12.00, 22.00), indicating a relatively high level of demoralization and the average HADS score in the SI group was 23.00 (19.00, 25.00) representing an elevated level of depression and anxiety. Other scores for specific dimensions of each scale are shown in Table 2.

**Table 1:** Descriptive statistics of sociodemographic characteristics and SIOSS scores of cancer patients (*N* = 588)

Variables	Cancer of patients, *N* (%)	SIOSS scores, median (IQR)	*Z/H*	*P* value
Gender			− 3.667^b^	< 0.001
Male	350 (59.5%)	7.00 (5.00,11.00)		
Female	238 (40.5%)	9.00 (6.00,12.00)		
Marital status			11.919^a^	0.003
Married	547 (93.0%)	8.00 (5.00,11.00)		
Single	27 (34.6%)	11.00 (9.00,14.00)		
Divorced or widowed	14 (2.4%)	10.64 ± 5.73		
Medical financial burden			50.392^a^	< 0.001
Not at all	37 (6.3%)	6.49 ± 3.28		
A little	236 (40.1%)	7.00 (5.00,9.00)		
Some	213 (36.2%)	9.00 (6.00,12.00)		
Extreme	102 (17.3%)	10.00 (7.00,14.00)		
Living condition			− 3.962^b^	< 0.001
Not live alone	547 (93.0%)	8.00 (5.00,11.00)		
Solitary	41 (7.0%)	11.00 (7.00,15.00)		
Religious belief			− 3.023^b^	0.003
Yes	63 (10.7%)	9.76 ± 3.64		
No	525 (89.3%)	8.00 (5.00,11.00)		
Residence			− 4.513^b^	< 0.001
Rural	263 (44.7%)	9.00 (6.00,12.00)		
Urban	325 (55.3%)	7.00 (5.00,10.00)		
Level of education			2.793^a^	0.593
Primary and below	200 (34.0%)	7.00 (5.00,12.00)		
Junior high school	190 (32.3%)	8.00 (5.00,11.00)		
Senior high school	143 (24.3%)	9.00 (6.00,11.00)		
Junior college	36 (6.1%)	9.00 ± 3.08		
Bachelor degree or above	19 (3.2%)	8.00 (6.00,12.00)		
Income (yuan per month)			7.549^a^	0.023
< 3000	97 (16.5%)	8.00 (5.00,12.00)		
3000–5000	297 (50.5%)	8.00 (6.00,12.00)		
< 5000	194 (33.0%)	7.00 (4.00,11.00)		
Caretaker			10.786^a^	0.013
Family member	510 (86.7%)	8.00 (5.00,11.00)		
Nursing worker	8 (1.4%)	12.25 ± 2.66		
Friend	9 (1.5%)	10.56 ± 3.57		
Oneself	61 (10.4%)	8.23 ± 3.77		
Working state			− 5.838^b^	< 0.001
Still working	211 (35.9%)	9.00 (7.00,12.00)		
Sick rest	377 (64.1%)	7.00 (4.50,11.00)		
Cancer type			43.203^a^	< 0.001
Nasopharyngeal cancer	13 (2.2%)	6.46 ± 2.47		
Cholangiocarcinoma	12 (2.0%)	8.92 ± 3.60		
Lung cancer	188 (32.0%)	7.00 (5.00,10.00)		
Liver cancer	22 (3.7%)	9.55 ± 3.32		
Colorectal cancer	140 (23.8%)	9.00 (6.00,13.00)		
Lymphoma	9 (1.5%)	7.67 ± 3.97		
Ovarian cancer	8 (1.4%)	9.50 ± 6.09		
Breast cancer	11 (1.9%)	9.27 ± 3.95		
Esophageal cancer	26 (4.4%)	8.00 (4.75,11.00)		
Stomach cancer	87 (14.8%)	9.78 ± 4.17		
Thymus cancer	8 (1.4%)	8.00 (8.00,10.50)		
Pancreatic cancer	8 (1.4%)	7.75 ± 3.62		
Cervical cancer	9 (1.5%)	8.56 ± 4.72		
Other cancer	47 (8.0%)	7.00 (5.00,11.00)		

^a^Kruskal-Wallis test

^b^Mann-Whitney test

*IQR*, interquartile range

**Table 2:** Scores of SIOSS, DS-II-C, and HADS in each dimension

Scales	SI			NSI		
	Max	Min	Total, median (IQR)	Max	Min	Total, median (IQR)
SIOSS^a^						
Gross score	19.00	12.00	14.00 (13.00,15.00)	11.00	1.00	7.00 (5.00,9.00)
Desperation	11.00	4.00	8.00 (7.00,9.00)	8.00	0.00	3.00 (2.00,4.00)
Optimism	5.00	0.00	3.00 (2.00,4.00)	5.00	0.00	1.00 (1.00,2.00)
Sleep	4.00	1.00	4.00 (3.00,4.00)	4.00	0.00	2.00 (1.00,3.00)
Dissimulation	3.00	0.00	2.00 (2.00,3.00)	3.00	0.00	3.00 (2.00,3.00)
DS-II-C^b^						
Gross score	32.00	0.00	18.00 (12.00,22.00)	30.00	0.00	7.00 (4.00,14.00)
Meaning and goals	16.00	0.00	8.00 (5.00,10.00)	16.00	0.00	3.00 (1.00,7.00)
Stress and coping	16.00	0.00	9.00 (7.00,11.50)	16.00	0.00	5.00 (3.00,8.00)
HADS^c^						
Gross score	34.00	1.00	23.00 (19.00,25.00)	31.00	0.00	15.00 (10.00,22.00)
Anxiety	20.00	1.00	12.00 (10.00,14.00)	18.00	0.00	8.00 (6.00,12.00)
Depression	15.00	0.00	11.00 (9.00,12.00)	18.00	0.00	7.00 (4.00,10.00)

*SI*, Suicidal ideation, scores of Self-rating Idea of Suicide Scale (SIOSS) ≥ 12; *NSI*, non-suicidal ideation, scores of Self-rating Idea of Suicide Scale (SIOSS) < 12; *Max*, the maximum of the scores; *Min*, the minimum of the scores; *IQR*, interquartile range

^a^Self-rating Idea of Suicide Scale (SIOSS)

^b^Chinese version of Demoralization Scale II (DS-II-C)

^c^Hospital Anxiety and Depression Scale (HADS)

### Influencing factors associated with SI

Univariate analysis of the sociodemographic features indicated that gender (*Z* =  − 3.667, *P* < 0.001), marital status (*H* = 11.919, *P* = 0.003), medical financial burden (*H* = 50.392, *P* < 0.001), living condition (*Z* =  − 3.962, *P* < 0.001), religious belief (*Z* =  − 3.023, *P* = 0.03), residence (*Z* =  − 4.513, *P* < 0.001), income (*H* = 7.549, *P* = 0.023), caretaker (*H* = 10.786, *P* = 0.013), working state (*Z* =  − 5.838, *P* < 0.001), and cancer type (*H* = 43.203, *P* < 0.001) were all associated with SI (Table 1). Spearman’s correlation test indicated that the DS-II-C score (*r* = 0.403, *P* < 0.01) and HADS score (*r* = 0.383, *P* < 0.01) correlated positively with SI (Table 3). The VIF ranged from 1.023 to 1.127 in multi-collinearity analysis indicating that there was no collinearity. Continuous variables (HADS score and DS-II-C score) were input as raw data. Binary variables (gender, living condition, religious belief, residence, and working state), and ordinal categorical variables (medical financial burden and income) were assigned. Nominal variables (marital status, caretaker, and cancer type) were set as dummy variables (Table 4). Multiple stepwise linear regression analysis indicated that the change in demoralization (*β* = 0.328, *P* < 0.001), medical financial burden (*β* = 0.195, *P* < 0.001), working state (*β* =  − 0.113, *P* = 0.001), living condition (*β* = 0.130, *P* < 0.001), marital status (married; *β* =  − 0.102, *P* = 0.004), residence (*β* =  − 0.106, *P* = 0.003), gender (*β* = 0.112, *P* = 0.001), caretaker (nursing workers; *β* = 0.087, *P* = 0.014), and cancer type (stomach cancer, colorectal cancer; *β* = 0.124, *P* < 0.001; *β* = 0.121, *P* = 0.001) could explain 32.1% of SI in this sample (*F* = 28.705, *P* < 0.001). Additional data from the multiple stepwise linear regression analysis are shown in Table 5.

**Table 3:** Correlations among demoralization, hospital anxiety and depression, age, and suicidal ideation

	SIOSS score ^a^	DS-II-C score ^b^	HADS score ^c^	Age
SIOSS score^a^	1			
DS-II-C score^b^	.403^**^	1		
HADS score^c^	.383^**^	.796^**^	1	
Age	.013	.113^**^	.097^*^	1

^**^P value < 0.01; *P value < 0.05

^a^Measured with Self-rating Idea of Suicide Scale (SIOSS)

^b^Measured with Chinese version of Demoralization Scale II (DS-II-C)

^c^Measured with Hospital Anxiety and Depression Scale (HADS)

**Table 4:** Assignment of the independent variables

Variables	Assignment
Gender	Male = 1, Female = 2
Marital status	Single as reference, X1 = Married (0,1), X2 = Divorced or widowed (0,1)
Medical financial burden	Not at all = 1, A little = 2, Some = 3, Extreme = 4
Living condition	Not live alone = 0, Solitary = 1
Religious belief	Yes = 1, No = 2
Residence	Rural = 1, Urban = 2
Income (yuan per month)	< 3000 = 1, 3000–5000 = 2, > 5000 = 3
Caretaker	Oneself as reference, X1 = Nursing workers (0,1), X2 = Friends (0,1), X3 = Family member (0,1)
Working state	Still working = 0, Sick rest = 1
Cancer type	Lung cancer as reference, X1 = Nasopharyngeal cancer (0,1), X2 = Cholangiocarcinoma (0,1), X3 = Liver cancer (0,1), X4 = Colorectal cancer (0,1), X 5 = Lymphoma (0,1), X6 = Ovarian cancer (0,1), X7 = Breast cancer (0,1), X8 = Esophageal cancer (0,1), X9 = Stomach cancer (0,1), X10 = Thymus cancer (0,1), X11 = Pancreatic cancer (0,1), X12 = Cervical cancer (0,1), X13 = Other cancer (0,1)
HADS score^a^	Old value input
DS-II-C score^b^	Old value input

^a^Measured with Hospital Anxiety and Depression Scale (HADS)

^b^Measured with Chinese version of Demoralization Scale II (DS-II-C)

**Table 5:** Results of multiple linear regression analysis

Variables	*B*	*SE*	*β*	*t*	*P* value
Constant	5.499	1.064		5.169	< 0.001
Demoralization score	0.175	0.019	0.328	9.437	< 0.001
Medical financial burden	0.942	0.172	0.195	5.465	< 0.001
Working state	− 0.954	0.298	− 0.113	− 3.201	0.001
Living condition	2.065	0.546	0.130	3.783	< 0.001
Marital status (married)	− 1.621	0.555	− 0.102	− 2.919	0.004
Residence	− 0.860	0.286	− 0.106	− 3.008	0.003
Gender	0.917	0.283	0.112	3.242	0.001
Caretaker (nursing workers)	3.040	1.228	0.087	2.476	0.014
Cancer type (stomach cancer)	1.408	0.401	0.124	3.506	< 0.001
Cancer type (colorectal cancer)	1.146	0.342	0.121	3.346	0.001

*R*^2^ = 0.332; adjusted *R*^2^ = 0.321; *F* = 28.705; *P* < 0.001

*B* unstandardized coefficients; *SE* standard error; *β* standardized coefficients

## Discussion

The present study investigated the prevalence of SI as determined by administering the SIOSS to cancer patients in China. Several factors were found to be significantly associated with SI.

### Incidence of SI

Approximately 24.7% of participants were identified as having SI, which supports the prevalence reported by a recent Spanish study (25.2%) [25] and a study involving Chinese cancer patients (26.3%) [26]. A research project conducted in the U.S. found that 40.38% and 31.92% of adolescents and older aged cancer patients, respectively, had a positive SI assessment [27]. A systematic review revealed that SI incidence ranged significantly from 0.7 to 46.3% [28], likely because of variability in the methods used to calculate the SI and the transitoriness and complexity of this psychological condition. While medical advancements have improved treatments for cancer and cancer-associated pain, the prevalence of SI remains high in this patient population. Anxiety, distress, and depression affect cancer patients both mentally and physically, contributing to an increased risk of SI. As a result of the lack of precise measurement tools, the true incidence of SI may be higher than what is reported. Early detection of SI among cancer patients should be prioritized.

### SI with demoralization

Demoralization is characterized by personal distress and a self-reported inability to cope as a result of hopelessness, lack of self-worth, and loss of meaning and goals [29, 30]. It is considered a mental state of perceived disability and is common among cancer patients [31, 32]. Indeed, almost one in five cancer patients experienced some degree of demoralization [33]. In a Portuguese study, 52.5% of terminally ill cancer patients reported to experience demoralization [34] and a study from Southern Europe found that 25.1% of cancer patients experienced demoralization [32]. Xu et al. described an important relationship between demoralization and SI among cancer patients [35]. The results of the current study were consistent with these findings. The SI group had moderate to high demoralization scores. Importantly, this study was the first to show that demoralization (*β* = 0.328, *P* < 0.001) was the only intrinsic factor that influenced SI. The effects of cancer on negative emotional states such as demoralization may trigger the development of SI and SI, in turn, further aggravate negative internal states. This vicious circle can promote the transformation of SI into suicidal behavior. Demoralization has been identified as a critical influencing factor of SI among cancer patients. Thus, detecting and relieving demoralization in cancer patients may help to reduce the incidence of SI. Fraguell-Hernando et al. showed that individual meaning-centered psychotherapy-palliative care (IMCP-PC) is a beneficial psychotherapy approach suitable for advanced cancer patients, which can contribute to significantly improving demoralization [36]. These findings highlight the importance of identifying demoralization in cancer patients and the need to develop suitable scales for clinical application, which allow early identification and intervention.

### Other factors associated with SI

Marital status, living condition, medical financial burden, working state, residence, gender, cancer type, and caretaker were other factors significantly associated with SI.

### Single and solitary

Solitary cancer patients had a higher-level risk of SI than non-solitary patients (*β* = 0.130, *P* < 0.001). Single cancer patients were also more likely to experience SI than married patients (*β* =  − 0.102, *P* = 0.004), revealing a weakness in the social support system. Family support is a vital component of this system, helping cancer patients to handle stressful events appropriately and serving as a protective factor for SI [37]. Spouses also play a critical role in daily care and provide mental inspiration for cancer patients that helps to relieve the psychological burden and reduce loneliness [38]. As a result, solitary and single cancer patients at higher risk of SI are likely to require more attention from clinical medical workers and social support from friends, colleagues, and medical staff. While not able to affect a patient’s marital status and living condition, clinical workers can prioritize providing support to cancer patients who lack social networks. In addition, the supportive function of clinical social workers should be granted additional prominence. Nearly one-third of the subjects reported that the group-therapy approach was beneficial for the improvement of emotional function and the establishment of social support networks [39]. Support groups and group-therapy approaches could be considered by clinical workers for cancer patients who are single or solitary.

### Medical financial burden and working state

Pharmacotherapy is the primary treatment for neoplasms. A Chinese survey conducted in 37 tertiary hospitals suggested that gastric cancer patients spend approximately $5368 on out-of-pocket expenses each year, accounting for 63.8% of the preceding year’s household income and leaving 79.2% of families with an unbearable medical financial burden [40]. Cancer patients who carry a medical burden (*β* = 0.195, *P* < 0.001) are at a higher risk for developing SI. Cancer leads to additional disease expenses in part because it occurs as an unanticipated event that alters the economic spending patterns of a family. Additionally, cancer patients on sick rest (*β* =  − 0.113, *P* = 0.001) have a higher incidence of SI than those who are still working likely because holding a job aids the individual’s sense of personal value and social image. In addition, sick rest abruptly tightens a person’s budget and increases their financial medical burden. This external pressure facilitates negative feelings and increases the risk of SI among cancer patients. Indeed, treatment expenses are both a psychological and physical burden on patients and contribute to an increase in extremely negative thoughts such as suicide. Financial problems experienced by cancer patients can be addressed by improving the quality of and access to national medical insurance, which has also been confirmed by a study in Taiwan [41].

### Residence

Cancer patients from urban areas experienced a lower SI prevalence than those from rural areas (*β* =  − 0.106, *P* = 0.003). This is likely because health care in cities is generally more well-developed than in the country, and patients in urban areas often have more medical knowledge, which that gives them a better understanding of their disease and treatment options, alleviating anxiety and strengthening their confidence in medicine. A cohort study suggested that patients from rural areas experienced significantly higher SMR than those living in urban regions [42]. Similarly, a study of suicide incidence among patients with head and neck cancer suggested that urban residents were at lower risk of SI than rural residents [43]. Thus, there is a need to develop methods for spreading medical information and providing a better medical environment for cancer patients who live in rural areas so that they have an accurate perception of the disease and their treatment options.

### Gender

There is conflicting evidence in the literature about whether male or female cancer patients are at higher risk of SI [44, 45]. In the current study, being female was a risk factor for SI (*β* = 0.112, *P* = 0.001). A study in the U.S. confirmed that the suicide rate among patients with gastric cancer was eight-times higher for females than males in the gender-matched population [46]. The sensitivity and vulnerability characteristics of females may explain this result. It suggested that the clinical staff should focus more on the psychological state of female cancer patients.

### Colorectal cancer and stomach cancer

Patients with colorectal cancer (*β* = 0.121, *P* = 0.001) and stomach cancer (*β* = 0.124, *P* < 0.001) reported a higher level of SI than lung cancer patients, the result that was distinct from former studies. Although patients with these cancer types have a poor prognosis, the characteristics of each cancer are different [47, 48]. Due to the universality of poor dietary habits, the morbidity and mortality of colorectal and stomach cancer are important concerns. Worldwide, colorectal (10.0%) and stomach (5.6%) cancers are commonly diagnosed cancers ranking among the top 5 of the GLOBOCAN 2020 estimation. Furthermore, colorectal (9.4%) cancer and stomach (7.7%) cancer are the leading causes of cancer death following lung cancer [49]. Malnutrition caused by gastric cancer symptoms such as poor appetite and food reflux is closely related to the low quality of life of patients [50]. Colorectal cancer patients, especially those with rectal cancer, are likely to require an ostomy that can permanently alter their diet and further impact on their quality of life [51]. Changes in bowel patterns may also lead to altered body images, social difficulties, and sexual dysfunction [52, 53]. Patients with a poor cancer prognosis should be prioritized for SI screening.

### Caretaker

Cancer patients who had a nursing worker (*β* = 0.087, *P* = 0.014) as a long-term caretaker were more likely to have SI than those who were able to engage in self-care. When patients are tended by nursing workers, this may represent an absence of social support, such as the loss or lack of support and company from family members. In China’s social system network, nursing workers are only trained to complete basic operations and many lack the nursing care skills of professional clinical nurses. Neglect of the patients’ psychological needs may contribute to misunderstanding and hopelessness. Inner feelings of stigma experienced by cancer patients and inadequate training of nursing workers may contribute to SI in cancer patients tended by nursing workers.

### Limitations and strengths

This study had some limitations. First, the cross-sectional study design made it difficult to establish any causal relationships among the findings. Thus, a longitudinal, prospective study is required to validate the results. Second, the study population only included patients ≥ 18 years of age from two tertiary A general hospitals; thus, the study population was unlikely to be entirely representative of all cancer patients in China. The present study had two important strengths. While the study was cross-sectional, the findings had important implications for those engaged in clinical work in oncology. In addition, the SIOSS was used because it considered Chinese culture and could most accurately predict SI.

## Conclusion

In this study, a sample of Chinese patients with mixed cancer types had a 24.7% incidence of SI. Demoralization, medical financial burden, marital status, living condition, working state, caretaker, residence, gender, and cancer type were factors that most impacted on SI, especially demoralization. Cancer patients who were single, solitary, on sick rest, or had nursing workers as primary long-term caretakers had higher rates of SI. Cancer patients living in urban areas had a lower risk of SI than those in rural areas. SI was higher in female than in male patients and was higher in those with colorectal and stomach cancer than lung cancer. These findings suggested that medical staff should be more aware of the social context of each individual cancer patient and should formulate a specific clinical intervention that considers the most impactful factors. Further studies are required to assess additional factors and develop potential clinical interventions.

## Data Availability

Study data can be obtained by contacting the corresponding authors with reasonable demands.
